# A Therapeutic Whole-Tumor-Cell Vaccine Covalently Conjugated with a TLR7 Agonist

**DOI:** 10.3390/cells11131986

**Published:** 2022-06-21

**Authors:** Huju Chi, Yue Hao, Xia Wang, Li Tang, Yongqiang Deng, Xianxiong Chen, Feng Gao, Ou Sha, Guangyi Jin

**Affiliations:** 1 School of Basic Medical Sciences, Health Science Center, Shenzhen University, Shenzhen 518000, China; 2150220123@email.szu.edu.cn (H.C.); xia.wang@szu.edu.cn (X.W.); 2 School of Pharmaceutical Sciences, International Cancer Center, Health Science Center, Shenzhen University, Shenzhen 518000, China; yuehao@szu.edu.cn; 3 College of Health Science and Environmental Engineering, Shenzhen Technology University, Shenzhen 518000, China; tangli@sztu.edu.cn; 4 School of Dentistry, Health Science Center, Shenzhen University, Shenzhen 518000, China; deng.yq@szu.edu.cn (Y.D.); gzcxx@szu.edu.cn (X.C.); gaofeng@szu.edu.cn (F.G.)

**Keywords:** whole-tumor-cell vaccine, toll-like receptor 7 agonist, cancer immunotherapy, 5-azacytidine, reparixin

## Abstract

A single-protein or -peptide vaccine is not sufficient to arouse immune responses in cancer therapy. A whole-tumor-cell vaccine with complete cancer cell antigens and all conformations elicits robust immune responses and is a promising method for the treatment of advanced malignant tumors. In this study, we used 5-azacitidine to stimulate B16-F10 melanoma cells to express toll-like receptor (TLR) 3 on the cell surface and then chemically linked SZU-106, a small-molecule TLR7 agonist, to the cell surface with a pegylated linker to produce a novel whole-tumor-cell vaccine, abbreviated as Aza-BFcell-106. The vaccine stimulated mouse splenic lymphocytes and bone marrow-derived dendritic cells to secrete cytokines, promoted the maturation of dendritic cells and enhanced the capability of dendritic cells to present antigens. In a mouse model of melanoma, the vaccine effectively inhibited tumor growth, decreased tumor volume and prolonged survival. Further combination of the vaccine with a chemokine inhibitor, reparixin, significantly increased the infiltration of CD4+ and CD8+ T cells into the tumor environment, while the antitumor effect was significantly enhanced. The whole-tumor-cell vaccine Aza-BFcell-106 induced immune-activating responses in both in vitro and in vivo experiments, indicating that this vaccine has great potential to treat advanced malignant tumors.

## 1. Introduction

Cancer vaccines are a promising treatment modality for patients with advanced malignant tumors [1,2,3,4,5]. The ideal therapeutic vaccine for cancer should be both preventive and curative. Treating tumor cells with physical, chemical, or radioactive methods and injecting them into the body for tumor treatment is the methodology used for whole-tumor-cell vaccination [6,7,8,9,10,11,12]. Typical tumor vaccines target one or several specific tumor antigens and cannot include all tumor cell information relevant to immunity, such as whole antigen sequences and conformations that are required for immunity pattern recognition, often resulting in limited effects [13,14]. In this study, we sought to construct a novel whole-tumor-cell vaccine that retained whole immunogenicity and triggered an ideal antitumor immune response.

Pattern recognition receptors (PRRs) on antigen-presenting cells (APCs) are stimulated via molecular signals. Pathogen-associated molecular patterns (PAMPs) can vary from oligonucleotides to bacterial cell wall components [15,16]. Many current cell-based immunotherapies include one type of PAMP that stimulates only one PRR, resulting in a partial immune response. In contrast, effective vaccines, such as the yellow fever vaccine, are composed of several signals that interact with multiple PRRs to elicit a robust immune response [17,18]. Combining target antigens with molecular immune agonists is an important aspect in effective vaccines. The chemical identity of a stimulating signal and its proximity to target antigens work in concert to elicit a specific immune response, such as the lipid anchoring of molecular signals on tumor cells, enhancing the immune response [19]. However, the covalent attachment of small-molecule toll-like receptor (TLR) agonists to cell surfaces to produce a vaccine has not been attempted. Reiner Strick et al. reported that azacitidine (Aza) could induce cancer cells to express TLR3 to enhance immune recognition [20].

Hence, in this study, we first used Aza to stimulate B16-F10 melanoma cells to express TLR3 on the cell surface and further linked it with SZU-106, a TLR7 agonist, to the cell surface with a pegylated linker. We sought to address the following issues: whether chemical modification of the tumor cell surface with PAMP molecules enhances antitumor immunostimulation and whether this synergistic combination with modular chemistry potentiates favorable immune polarization. We showed that PAMP-labeled cells upregulated cell surface marker expression, which is critical for T cell activation. The constructs labeled with multiple PAMP molecules also modulated cytokine production, allowing for the potential to design targeted vaccines. We also observed macrophagocytosis of the PAMP-labeled cells, indicating a potential mechanism by which the immune-stimulating constructs are presented to endosomal TLR7. The covalent attachment of TLR7 agonists to the surfaces of tumor cells enhanced dendritic cell (DC) activation to induce the more specific recognition of tumor cells. Our approach demonstrates the significance of chemically conjugating PAMPs to target-cell antigens and using multiple PAMPs to develop more effective antitumor vaccines.

## 2. Materials and Methods

### 2.1. Cell Culture and Reagents

Mouse B16-F10 melanoma cells were purchased from the Institute of Biochemistry and Cell Biology of the Chinese Academy of Science (Shanghai, China) and cultured in DMEM (Invitrogen, Carlsbad, CA, USA) containing 10% fetal bovine serum (Thermo Fisher Scientific, Waltham, MA, USA) in a humidified atmosphere containing 5% CO2 at 37 °C. HEK-BLUE hTLR7 cells were purchased from InvivoGen (San Diego, CA, USA) and maintained in selective DMEM supplemented with 10 μg/mL blasticidin (Thermo Fisher Scientific, USA) and 100 μg/mLZeocin (San Diego, CA, USA). 5-Aza was purchased from Apexbio Technology LLC (Houston, TX, USA), polyinosinic–polycytidylic acid (poly: IC) was purchased from Sigma (St. Louis, MO, USA), and reparixin was obtained from MedChem Express LLC (Princeton, NJ, USA).

### 2.2. Synthesis of the TLR7 Agonist SZU-106

**Figure 1:**
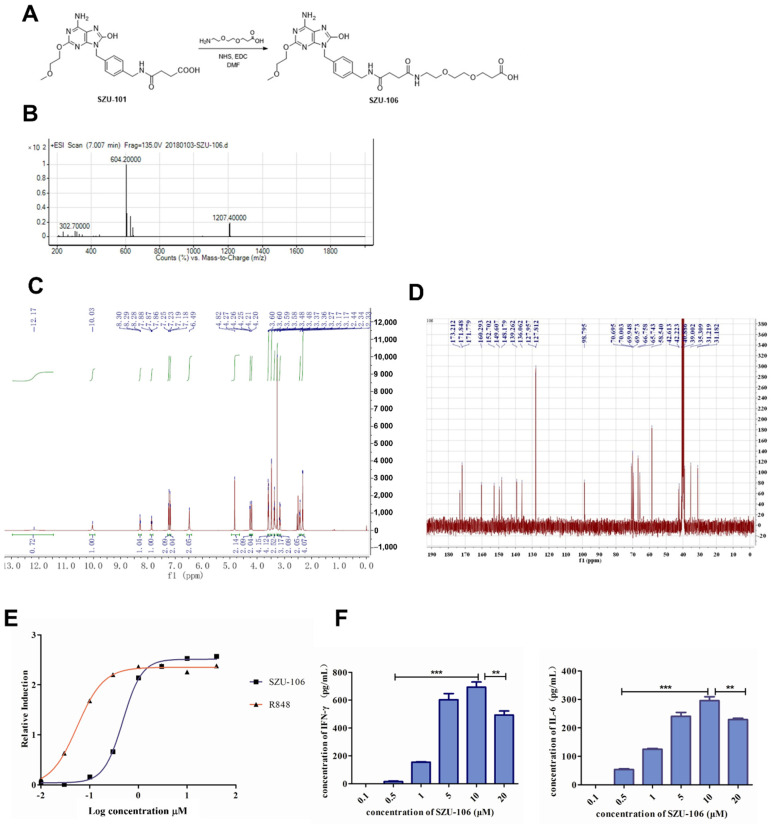
Synthesis of SZU-106 and the activation of TLR7 by SZU-106. (**A**). The synthesis of SZU-106 from SZU-101 is shown. (**B**–**D**). Structure confirmation for SZU-106 was performed with mass spectroscopy, 1H NMR, and 13C NMR. (**E**). SZU-106 selectively activated TLR7 and increased the expression of SEAP. (**F**). Cytokine induction in mouse splenic lymphocytes by the TLR7 agonist SZU-106 was evaluated. Data are expressed as the mean ± SD (*n* = 6) of one representative experiment. ** *p* < 0.01; *** *p* < 0.001.

A small-molecule TLR7 agonist, SZU-106, was synthesized from SZU-101 (Figure 1) [18]. An equivalent of SZU-101 was dissolved in 2 mL of DMF together with 1.2 equivalents of *N*-hydroxysuccinimide (NHS) and 1.3 equivalents of *N*-(3-dimethylaminopropyl)-*N*′- ethylcarbodiimide hydrochloride (EDC). The mixture was stirred at room temperature for 4 h in a nitrogen atmosphere. Then, 3-[2-(2-Aminoethoxy)ethoxy]-propanoic acid (1 equivalent) was added to the mixture. The reaction mixture was allowed to stir overnight at room temperature. The final product was obtained by precipitation in cold water and subsequent purification by column chromatography (CH_2_C_l2_:CH_3_OH = 20:1). ^1^H NMR (400 MHz, DMSO-d6) δ; ^13^C NMR (101 MHz, DMSO-d6) δ 173.21, 171.85, 171.78, 160.29, 152.70, 149.61, 148.18, 139.26, 136.06, 127.96, 127.81, 98.80, 70.70, 70.00, 69.95, 69.57, 66.76, 65.74, 58.74, 42.61, 42.23, 40.88, 35.31, 31.22, 31.18. MS (ESI): 604.20 [M + H]+.

### 2.3. HEK-BLUE Assay

HEK-BLUE hTLR7 cells were purchased from InvivoGen (San Diego, CA, USA). The cells stably expressed human TLR7 and a SEAP reporter, which can be used to detect TLR7 agonism through the activation of NF-kB signaling. The cells were maintained in selective DMEM supplemented with 10 μg/mL blasticidin and 100 μg/mL Zeocin™. After incubation with different doses of SZU-106, the cells were tested using a HEK-BLUE detection kit, according to the manufacturer’s instructions. The TLR7 agonist imiquimod and SZU-101 were used as positive controls. The experiments were repeated 3 times. The induction of TLR7 activation was visualized and assessed by measuring the OD value at 620–655 nm.

### 2.4. Cytokine Enzyme-Linked Immunosorbent Assay (ELISA)

The levels of cytokines secreted into the medium of immunocytes were measured and repeated three times by ELISA (Sigma, Ronkonkoma, NY, USA), according to the manufacturer’s instructions. In brief, 100 μL of sample solution was added to each well of a 96-well plate. After incubation for 120 min, the plate was incubated with a biotinylated antibody. Immunoreactivity was determined using an avidin-HRP-TMB detection system. The reactions were stopped by the addition of a TMB buffer, and absorbance was measured at 450 nm using a microplate reader. A curve of the absorbance vs. standard-well concentrations was plotted. The concentrations of cytokines were determined from the absorbance of the samples by using the standard curve.

### 2.5. Lymphocyte Isolation and Generation of Bone Marrow-Derived DCs (BMDCs)

Primary splenocytes were isolated from the spleens of C57BL/6 mice using a lymphocyte separation liquid. The splenocytes were cultured in RPMI complete medium containing 10% fetal bovine serum. Bone marrow cells were first isolated from the femurs and tibias of C57BL/6 mice, following previous studies [21]. The obtained cells were seeded in 24-well culture plates in RPMI complete medium. To induce DC differentiation, 20 ng/mL granulocyte macrophage colony-stimulating factor (GM-CSF; PeproTech, Cranbury, NJ, USA) and 10 ng/mL interleukin (IL)-4 (PeproTech, Cranbury, NJ, USA) were added. All cultures were fed by replacing half of the medium and cytokines on days 3 and 5. On day 7, the cells were collected for further use.

### 2.6. Mouse Model of Melanoma

Female C57BL/6 mice (4–6 weeks old, 18–22 g; Laboratory Animal Center, Guangzhou, China) were maintained on a 12/12 h light/dark cycle, with food and water available ad libitum. B16-F10 cells (1 × 10^5^) were resuspended in phosphate-buffered saline (PBS) and subcutaneously injected into the right back of the mice. Tumor growth was measured with a caliper using the ellipse surface formula (Length × Width). When the tumor reached 5 mm in size, we started treatment with a vaccine (s.c., once a week) and reparixin (15 mg/kg, s.c., once every two days). The experiments were repeated 3 times. All procedures were conducted in strict adherence to the Guide for the Care and Use of Laboratory Animals and were approved by the Animal Care and Use Committee of the Health Science Center, Shenzhen University.

### 2.7. Flow Cytometry

Antibodies specific for CD11c (#117301), CD80 (#104735), CD86 (159202), and CD40 (102802) were purchased from BioLegend (San Diego, CA, USA). BMDCs were harvested on day 7 and cocultured with the Aza-BFcell-106 vaccine for 24 h. The BMDCs were subsequently collected, stained with antibodies for 20 min, and then detected with a flow cytometer (BD BioScience, NJ, USA). The experiments were repeated 3 times.

### 2.8. Preparation of a Whole-Cell Vaccine

SZU-106 (1 mg, 1.66 μmol) was added to a suspension of NHS (2 μmol) and EDC (2 μmol) in 166 μL of DMF. After stirring at room temperature for 18 h, the resulting conjugate was confirmed using liquid chromatography–mass spectroscopy (LC-MS) and diluted with a PBS buffer as a solution of SZU-106-NHS. B16-F10 cells were treated with 0.5 μM Aza for 72 h while in the log-growth phase, with the medium and drug changed every 24 h. The cells were collected after drug treatment, centrifuged, and washed with the PBS buffer. A solution of SZU-106-NHS (50 μM, 360 μL) in the PBS buffer was incubated with the cells (1 × 10^7^) in a shaker for 1 h at room temperature. After the coupling was completed, the supernatant was discarded by centrifugation, and the cells were repeatedly washed using PBS. Aza-treated B16-F10 cells were conjugated with SZU-106 and then killed by UV light exposure (10.8 J/cm^2^ for 10 min) to prepare a whole-cell vaccine named Aza-BFcell-106.

### 2.9. Cell Viability Assay

The cell viability of melanoma cell lines was analyzed three times with Cell Counting Kit-8 (CCK-8; MCE), according to the manufacturer’s instructions. In brief, B16-F10 cells were digested with 0.25% trypsin, seeded in a 96-well plate at a density of 8000 cells/well, and incubated for 24 h. The cells were treated with different concentrations of Aza. After stimulation for 24 h, each well of the plate was treated with 10 μL of CCK-8 solution and then incubated for 2–4 h. Absorbance was measured at 450 nm using a microplate reader.

### 2.10. Western Blots

B16-F10 cells were treated with 0.5 μM Aza. After 24 h, the cells were harvested, washed twice with PBS, and then lysed with 200 μL of lysis buffer (20 mM Tris-HCl, pH 8.0; 10% glycerol; 5 mM MgCl_2_; 0.15 M KCl; and 0.1% Nonidet P-40 protease inhibitor). After determining protein concentrations, the cell lysates were subjected to electrophoresis using 10% SDS-PAGE gels and transferred to PVDF membranes. The membranes were incubated with primary and secondary antibodies with proper blocking procedures and finally exposed to X-ray film. Rabbit anti-mouse β-actin and TLR3 antibodies were used as the primary antibodies, and goat anti-rabbit IgG-HRP was used as the secondary antibody. Each experiment was repeated 3 times. All of the antibodies were purchased from Cell Signaling Technology (CST, Danvers, MA, USA).

### 2.11. Immunohistochemical (IHC) Staining

Fresh tumor tissue samples were obtained from treated mice and were fixed immediately in 10% neutral buffered formalin. The fixed tumor tissue samples were dehydrated in an ethanol gradient, cleared in pure xylene, and embedded in paraffin blocks. Sections (3 μm) were cut and mounted onto slides. Each tissue was used to prepare 3 slides for IHC analysis. The sections were deparaffinized, rehydrated in xylene and ethanol, and then depigmented with 1% oxalic acid and 0.5% potassium permanganate. IHC staining (DAB, CST) was performed according to the manufacturer’s instructions, after antigen retrieval. Briefly, the sections were incubated with primary antibodies overnight and with a secondary antibody for 30 min. Rabbit anti-mouse CD4 (#48274) and CD8 (#85336) antibodies were used as the primary antibodies, and goat anti-rabbit IgG-HRP was used as the secondary antibody (CST). Subsequently, the slides were covered with one drop of DAB Chromogen Concentrate for visualization. After immunostaining, the slides were counterstained with hematoxylin, and a cover slip was mounted with an aqueous mounting medium. Each experiment was repeated 3 times.

### 2.12. Cytometric Bead Array Assay

Serum was collected at 2, 4, 8, 12, 24, and 48 h after Aza-BFcell-106 vaccine injection or PBS injection. Samples were diluted 1:4 with an assay buffer and evaluated using a cytometric bead array (CBA) system, following the manufacturer’s instructions (BD BioScience, Franklin Lakes, NJ, USA). The experiments were repeated 3 times.

### 2.13. DC Phagocytic Function Test

BMDCs were harvested on day 7 and stained with 10 μM cell membrane dye-Dil for 20 min. Meanwhile, B16-F10 cells were collected, coupled with SZU-106, and subsequently stained with 10 μM cell membrane dye-Dio for 20 min. The stained BMDCs and B16-F10 cells were washed twice with PBS separately and then cocultured in a small dish for 2–4 h so that the BMDCs could phagocytose the B16-F10 cells. The cells were then stained with DAPI and transferred to slides. The slides were observed with an inverted laser confocal microscope, and images were acquired under identical acquisition settings. The experiments were repeated 3 times.

### 2.14. Statistical Analysis

Data are presented as the mean ± standard deviation (SD) of one representative experiment. Differences between groups were analyzed by analysis of variance (ANOVA) using a one-way or mixed factorial design as appropriate, followed by Fisher’s least significant difference (LSD) tests or simple-effects ANOVA. All statistical analyses were performed using GraphPad Prism 6 software (version 6.02, La Jolla, CA, USA). Significance was defined as *p* < 0.05.

## 3. Results

### 3.1. Synthesis of SZU-106 and the Activation of TLR7 by SZU-106

The TLR7 agonist SZU-106 was synthesized from SZU-101, as shown in Figure 1A. The structure was confirmed by 1H NMR, 13C NMR, and mass spectroscopy (Figure 1B–D). To validate that SZU-106 is an agonist of TLR7, a HEK-Blue™ hTLR7 reporter assay was performed. The results confirmed that SZU-106 selectively activated TLR7 and increased the expression of SEAP by activating the NF-κB pathway. When the concentration of SZU-106 reached 10 μM, the activation of TLR7 by SZU-106 was more profound than that induced by another TLR7 agonist, R848 (*p* < 0.001; Figure 1E). The effect of SZU-106 on the production of cytokines was also examined in mouse splenic lymphocytes. As shown in Figure 1F, SZU-106 at a concentration of 0.5–10 μM increased the release of interferon (IFN)-γ and IL-6 in a dose-dependent manner after 24 h of treatment with SZU-106.

### 3.2. Design and Preparation of the Whole-Tumor-Cell Vaccine

**Figure 2:**
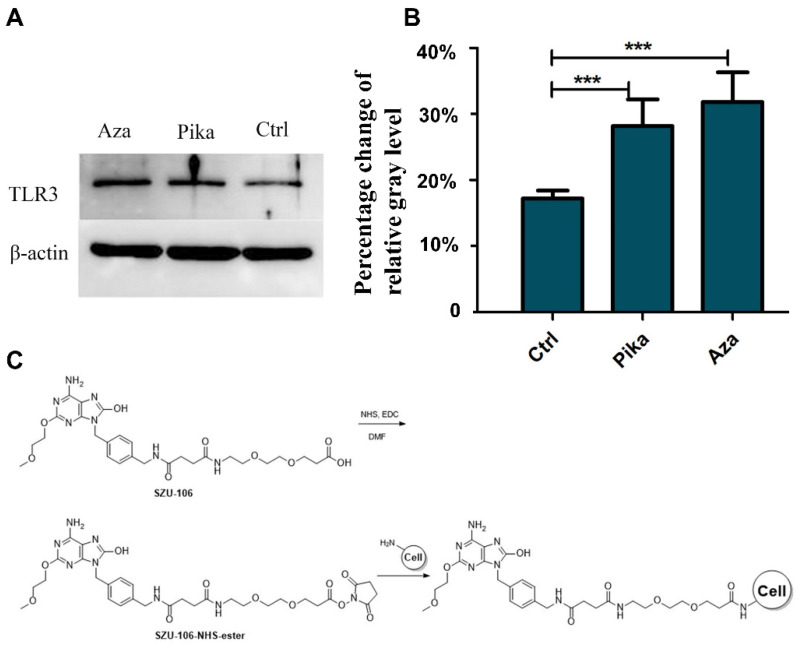
Design and preparation of the whole-tumor-cell vaccine. (**A**,**B**). Effect of Aza on the expression level of TLR3 in B16-F10 cells. (**A**) shows representative Western blots for TLR3 and the loading control (β-actin) in each group. (**B**) is a bar graph showing the mean ± SD of the percentage change from control TLR3 expression. Data are expressed as the mean ± SD (*n* = 3) of one representative experiment. The same results were obtained in three replicates. *** *p* < 0.001. (**C**) Scheme illustrating the conjugation of SZU-106 to mouse B16-F10 melanoma cells.

To modify cell surfaces with PAMPs, we first used Aza to stimulate B16-F10 melanoma cells to express TLR3 on their cell surface. Three days after treatment with Aza, total protein was extracted from the B16-F10 cells, and Western blotting analysis was performed. The results confirmed that, compared with the control treatment, Aza at a concentration of 0.5 μM induced the significant upregulation of TLR3 expression in B16-F10 cells (*p* < 0.001; Figure 2A,B). Aza-treated B16-F10 cells were then conjugated with SZU-106, which has a pegylated chain and terminal carboxylate to react with free amines on cell surfaces (Figure 2C). The cells were then inactivated with UV light to prepare a chemically modified whole-cell vaccine, Aza-BFcell-106.

### 3.3. Aza-BFcell-106 Triggered Cytokine Release In Vitro

**Figure 3:**
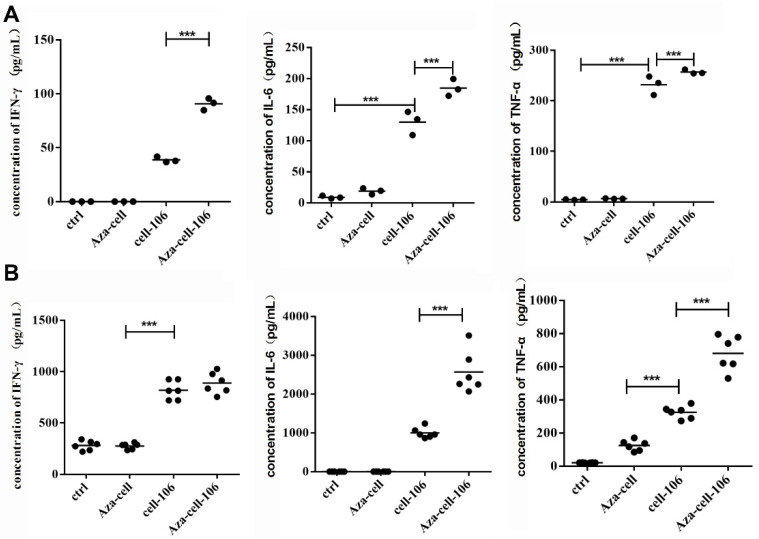
Cytokine release induced by Aza-BFcell-106 in vitro. (**A**). Aza-BFcell-106 stimulated the secretion of IFN-γ, IL-6, and TNF-α by splenic lymphocytes. (**B**). Aza-BFcell-106 stimulated the secretion of IFN-γ, IL-6, and TNF-α by BMDCs. Data are expressed as the mean ± SD (*n* = 6) of one representative experiment. The same results were obtained in three replicate experiments. *** *p* < 0.001.

Murine splenic lymphocytes and BMDCs were isolated and cocultured with BF cells, Aza-BF cells, BFcell-106, or Aza-BFcell-106. After 24 h of coculture, Aza-BFcell-106 stimulated the most profound secretion of IFN-γ, IL-6, and TNF-α by the splenic lymphocytes (*p* < 0.001; Figure 3A) and BMDCs (*p* < 0.001; Figure 3B). BFcell-106 stimulated the secretion of IFN-γ, IL-6, and TNF-α by the splenic lymphocytes and BMDCs as well, but to a lesser extent than Aza-BFcell-106 (*p* < 0.001; Figure 3). BFcell and Aza-BFcell did not evoke cytokine release from the splenic lymphocytes or BMDCs (*p* > 0.05; Figure 3). These findings suggest that the release of cytokines from splenic lymphocytes or BMDCs induced by Aza-BFcell-106 may depend on the presence of SZU-106, which was conjugated to the BF cells.

### 3.4. Aza-BFcell-106 Promoted the Maturation of DCs without Affecting DC Phagocytosis In Vitro

**Figure 4:**
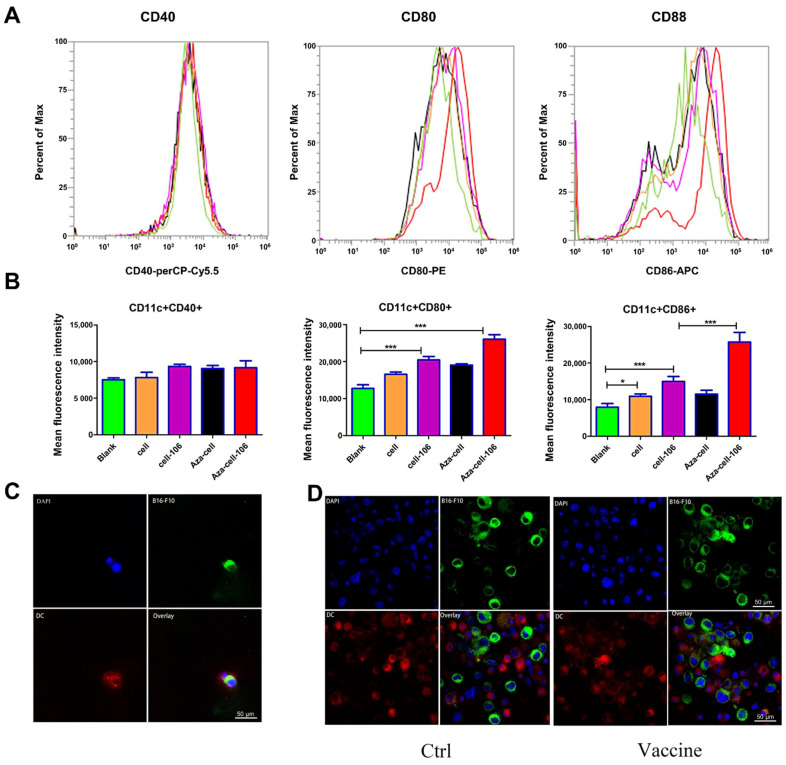
Aza-BFcell-106 promoted the maturation of BMDCs without affecting BMDC phagocytosis. (**A**). The peak map shows that the peak for Aza-cell-106 is shifted to the right of the peak for the blank group. (**B**). Average fluorescence intensity was analyzed from the corresponding peak map. The colors representing the different groups were kept consistent between the peak map in (**A**) and the data in (**B**). The graph shows the mean ± SD value (*n* = 3) of one representative experiment. The same results were obtained in three replicate experiments. * *p* < 0.05; *** *p* < 0.001. (**C**,**D**). DCs (red) phagocytosed B16-F10 cells (green) conjugated with SZU-106. (**C**) shows the phagocytic function of a single DC. (**D**) shows the phagocytic function of DCs in the control group and Aza-BFcell-106 group. Blue indicates DAPI staining.

The expression levels of CD40, CD80, and CD86 on BMDCs were tested by flow cytometry analysis to determine the degree of BMDC maturation after 24 h of coculture with Aza-BFcell-106. As the results show, coculture with Aza-BFcell-106 significantly increased the expression levels of CD80 and CD86, whereas the expression level of CD40 showed no difference among the groups (Figure 4A,B). The results suggested that coculture with Aza-BFcell-106 promoted the maturation of BMDCs.

Phagocytic function was tested to determine whether DCs phagocytose B16-F10 cells after SZU-106 conjugation. The results showed that SZU-106 conjugation to B16-F10 cells did not influence DC phagocytosis (Figure 4C,D). Figure 4C shows the phagocytosis performed by a single DC. Figure 4D shows the phagocytosis by DC cells in a single field of view.

### 3.5. Antitumor Effect of Aza-BFcell-106 and the Combination of Aza-BFcell-106 and Reparixin In Vivo

**Figure 5:**
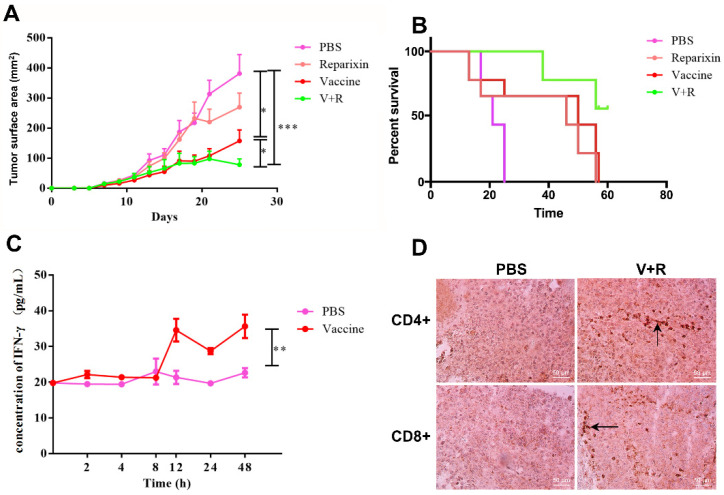
In vivo antitumor effects of Aza-BFcell-106 and a combination of Aza-BFcell-106 and reparixin. (**A**) Tumor volumes and (**B**) survival curves of C57BL/6 mice implanted subcutaneously with melanoma cells are shown. (**C**) IFN-γ levels were measured at 2, 4, 8, 12, 24, and 48 h post-treatment with Aza-BFcell-106. Data are shown as the mean ± SD (*n* = 6) of one representative experiment. (**D**) T cells (arrows) infiltrating into tumor tissues after Aza-BFcell-106 treatment are shown. Representative images show the staining for CD4 and CD8. The intensity of CD4 and CD8 staining was also measured. * *p* < 0.05; ** *p* < 0.01; *** *p* < 0.001.

Consistent with the in vitro results, Aza-BFcell-106 alone significantly decreased tumor growth. Treatment with a combination of Aza-cell-106 and reparixin led to more significant tumor shrinkage. The tumor size in the combination group remained stable over 15–25 days after the implantation of cancer cells (Figure 5A). In addition, the combination of Aza-cell-106 and reparixin significantly prolonged the survival period of the tumor-implanted mice. Mice in the control group began to die on day 14, and all mice in the control group died within 22 days, whereas mice in the group treated with the combination of Aza-cell-106 and reparixin survived till day 60. The survival curves are shown in Figure 5B.

Compared with the control treatment, the Aza-BFcell-106 vaccine induced a significant increase in IFN-γ release into the serum at 12 h post-treatment in melanoma-bearing mice (Figure 5C). To detect lymphocyte infiltration, mice were sacrificed, and tumor tissue samples were collected and analyzed by IHC staining with anti-CD4 and anti-CD8 antibodies. The tumors from the Aza-BFcell-106 group showed strong positive IHC staining for CD4 and CD8, indicating that Aza-BFcell-106 could largely promote T lymphocyte infiltration (Figure 5D).

## 4. Discussion

Low concentrations of Aza can activate the expression of endogenous retroviruses (ERVs) silenced in tumor cells by methylation, thus activating immunity through the virus defense pathway [22]. We found that low concentrations of Aza could also effectively stimulate splenic lymphocytes to produce immune cytokines. It has been shown that Aza and Pika (poly: IC derivatives) can activate the downstream NF-κB signaling pathway by interacting with TLR3, thereby stimulating the production of immune factors such as IFN [22,23]. Our Western blotting data confirmed that Aza indeed enabled the high expression of TLR3 in tumor cells. TLRs recognize PAMPs and mediate the production of cytokines necessary for effective immunization. TLR7 recognizes single-stranded RNA and synthetic small molecules. In this experiment, a novel whole-tumor-cell vaccine was prepared by coupling SZU-106, a small-molecule TLR7 agonist, with Aza-stimulated B16-F10 melanoma cells.

In preparing the Aza-BFcell-106 vaccine from inactivated tumor cells, we first attempted to inactivate tumor cells by 4% paraformaldehyde fixation; however, this approach did not achieve the desired effect in in vivo or in vitro experiments. Although the morphological structure of the cells is well maintained with paraformaldehyde fixation, the principal action of this fixation is the chemical cross-linking of extracellular amino residue groups, which reduces the antigenicity of certain intracellular components. After a series of attempts and preliminary experiments, we finally decided to inactivate and kill tumor cells by UV irradiation. In addition, tumor cells were completely washed before and after coupling so that the whole-tumor-cell vaccine did not contain residual Aza or SZU-106 monomers.

To explore the effects of the prepared Aza-BFcell-106 vaccine, we first performed a series of in vitro experiments. Splenic lymphocytes and BMDCs were stimulated with Aza-BFcell-106. The data showed that, compared with Aza-BFcell and SZU-106-BFcell, Aza-BFcell-106 could stimulate immune cells to produce significant quantities of cytokines, indicating that the Aza-BFcell-106 vaccine was more immunogenic than the other constructs. The cytokines IL-6 and IFN-γ play important roles in immune regulation. IFN-γ is mainly involved in cellular immunity and is an activator of macrophages. It also promotes the maturation, proliferation, and differentiation of CD8+ cytotoxic T lymphocytes. IFN-γ is the only type II IFN that inhibits tumor cell proliferation and has antiviral and antitumor effects. IL-6 is primarily involved in humoral immunity and antibody production. IL-12 is a T cell stimulator that mediates the cytotoxic activity of NK cells and CD8+ cytotoxic T lymphocytes and plays a key role in antitumor immunity. Therefore, IL-6 and IFN-γ were used as indicators for evaluating immune activation in this experiment.

The role of a vaccine depends on the ability of the vaccine to be recognized and presented by APCs. DCs are the most powerful APCs. DCs can take tumor antigens from tumor vaccines and process them into epitopes. Therefore, we used mouse BMDCs as a model to study immunity in vitro. We observed that CD86 and CD80, costimulatory factors on the surfaces of DCs, were highly expressed. The high expression of CD86 and CD80 indicated that DC maturity was enhanced, and antigen presentation abilities were greatly elevated.

To verify the therapeutic effect of Aza-BFcell-106, we subcutaneously injected the Aza-BFcell-106 vaccine or other constructs into melanoma-bearing mice. Compared with that of the tumors in the PBS group, the growth rate of the tumors in the vaccine-injected group was significantly slower. When Aza-BFcell-106 was combined with the chemokine inhibitor reparixin, tumor size was further suppressed, and the survival rate was 100% until the 30th day. During the experiment, the combination group survived normally, and their body weight did not show a significant decrease. From the mouse tumor model, it can be seen that the Aza-BFcell-106 vaccine is a promising immunotherapy that deserves further exploration and application research.

Dan Lu et al. reported that calreticulin could be unregulated by the demethylating agent 5-aza-2′-deoxycytidine and Aza could achieve the same effect [24,25]. Our Aza-BFcell-106 vaccine obviously could not exclude such additional effects. It has been reported that the CXCR1/CXCR2 axis regulates myeloid-derived suppressor cells (MDSCs) in the tumor environment [26]. Indeed, the antitumor effect of the Aza-BFcell-106 vaccine was increased dramatically when combined with the CXCR1/CXCR2 inhibitor reparixin, which is in clinical trials [26]. Our data showed that Aza-cell-106 combined with reparixin increased the amounts of CD4+ T cells and CD8+ T cells in the tumor tissue, indicating that the therapeutic effect of Aza-cell-106 was enhanced by regulating the tumor immune microenvironment.

## 5. Conclusions

In conclusion, we have designed and completed a proof-of-concept study of a novel whole-tumor-cell vaccine made by chemically modifying the cell surface of cancer cells, which resulted in a powerful antitumor effect. Based on our experimental data and the relevant literature, we speculate that the Aza-BFcell-106 vaccine is multifunctional. Considering the complicated and time-consuming process of creating personalized antitumor vaccines [27,28], the Aza-BFcell-106 vaccine can be produced conveniently in less than 10 days by separating personal cancer cells from the biopsy sample of each patient, which would be favorable for patients and practical for clinical application.

## Data Availability

Not applicable.

## Abbreviations

PRRs: pattern recognition receptors; APCs: antigen-presenting cells; PAMPs: pathogen-associated molecular patterns; TLR: toll-like receptor; Aza: azacitidine; DC: dendritic cell; ELISA; cytokine enzyme-linked immunosorbent assay; BMDCs: bone marrow-derived DCs; GM-CSF: granulocyte macrophage colony-stimulating factor; IL: interleukin; LC-MS: liquid chromatography–mass spectroscopy; IHC: immunohistochemical; CBA: cytometric bead array; SD: standard deviation; LSD: least significant difference; IFN: interferon; MDSCs: myeloid-derived suppressor cells.

## References

[1] Thomas S., Prendergast G.C. (2016). Cancer Vaccines: A Brief Overview. Methods Mol. Biol..

[2] Hochnadel I., Kossatz-Boehlert U., Jedicke N., Lenzen H., Manns M.P., Yevsa T. (2017). Cancer vaccines and immunotherapeutic approaches in hepatobiliary and pancreatic cancers. Hum. Vaccines Immunother..

[3] Pan R.Y., Chung W.H., Chu M.T., Chen S.J., Chen H.C., Zheng L., Hung S.I. (2018). Recent Development and Clinical Application of Cancer Vaccine: Targeting Neoantigens. J. Immunol. Res..

[4] Saxena M., van der Burg S.H., Melief C.J.M., Bhardwaj N. (2021). Therapeutic cancer vaccines. Nat. Rev. Cancer.

[5] Perez C.R., De Palma M. (2019). Engineering dendritic cell vaccines to improve cancer immunotherapy. Nat. Commun..

[6] Copier J., Dalgleish A. (2006). Overview of tumor cell-based vaccines. Int. Rev. Immunol..

[7] de Gruijl T.D., van den Eertwegh A.J., Pinedo H.M., Scheper R.J. (2008). Whole-cell cancer vaccination: From autologous to allogeneic tumor- and dendritic cell-based vaccines. Cancer Immunol. Immunother..

[8] Kudrin A., Hanna M.G. (2012). Overview of the cancer vaccine field: Are we moving forward?. Hum. Vaccines Immunother..

[9] Chiang C.L., Benencia F., Coukos G. (2010). Whole tumor antigen vaccines. Semin. Immunol..

[10] Chiang C.L., Kandalaft L.E., Coukos G. (2011). Adjuvants for enhancing the immunogenicity of whole tumor cell vaccines. Int. Rev. Immunol..

[11] Aikins M.E., Xu C., Moon J.J. (2020). Engineered Nanoparticles for Cancer Vaccination and Immunotherapy. Acc. Chem. Res..

[12] Korbelik M. (2021). Optimization of Whole Tumor Cell Vaccines by Interaction with Phagocytic Receptors. Vaccines.

[13] Geng F., Guo J., Guo Q.Q., Xie Y., Dong L., Zhou Y., Liu C.L., Yu B., Wu H., Wu J.X. (2019). A DNA vaccine expressing an optimized secreted FAPα induces enhanced anti-tumor activity by altering the tumor microenvironment in a murine model of breast cancer. Vaccine.

[14] Sharma M., Krammer F., Garcia-Sastre A., Tripathi S. (2019). Moving from Empirical to Rational Vaccine Design in the ‘Omics’ Era. Vaccines.

[15] Akira S., Takeda K., Kaisho T. (2001). Toll-like receptors: Critical proteins linking innate and acquired immunity. Nat. Immunol..

[16] Satoh T., Akira S. (2016). Toll-like Receptor Signaling and Its Inducible Proteins. Microbiol. Spectr..

[17] Staples J.E., Bocchini J.A., Rubin L., Fischer M. (2015). Yellow Fever Vaccine Booster Doses: Recommendations of the Advisory Committee on Immunization Practices, 2015. MMWR Morb. Mortal. Wkly. Rep..

[18] Zhu J., He S., Du J., Wang Z., Li W., Chen X., Jiang W., Zheng D., Jin G. (2015). Local administration of a novel Toll-like receptor 7 agonist in combination with doxorubicin induces durable tumouricidal effects in a murine model of T cell lymphoma. J. Hematol. Oncol..

[19] McHugh R.S., Ahmed S.N., Wang Y.C., Sell K.W., Selvaraj P. (1995). Construction, purification, and functional incorporation on tumor cells of glycolipid-anchored human B7-1 (CD80). Proc. Natl. Acad. Sci. USA.

[20] Strick R., Strissel P.L., Baylin S.B., Chiappinelli K.B. (2016). Unraveling the molecular pathways of DNA-methylation inhibitors: Human endogenous retroviruses induce the innate immune response in tumors. Oncoimmunology.

[21] Poon G.F., Dong Y., Marshall K.C., Arif A., Deeg C.M., Dosanjh M., Johnson P. (2015). Hyaluronan Binding Identifies a Functionally Distinct Alveolar Macrophage-like Population in Bone Marrow-Derived Dendritic Cell Cultures. J. Immunol..

[22] Chiappinelli K.B., Strissel P.L., Desrichard A., Li H., Henke C., Akman B., Hein A., Rote N.S., Cope L.M., Snyder A. (2015). Inhibiting DNA Methylation Causes an Interferon Response in Cancer via dsRNA Including Endogenous Retroviruses. Cell.

[23] Ohtani H., Ørskov A.D., Helbo A.S., Gillberg L., Liu M., Zhou W., Ungerstedt J., Hellström-Lindberg E., Sun W., Liang G. (2020). Activation of a Subset of Evolutionarily Young Transposable Elements and Innate Immunity Are Linked to Clinical Responses to 5-Azacytidine. Cancer Res..

[24] De Beck L., Melhaoui S., De Veirman K., Menu E., De Bruyne E., Vanderkerken K., Breckpot K., Maes K. (2018). Epigenetic treatment of multiple myeloma mediates tumor intrinsic and extrinsic immunomodulatory effects. Oncoimmunology.

[25] Feng D., Gip P., McKenna K.M., Zhao F., Mata O., Choi T.S., Duan J., Sompalli K., Majeti R., Weissman I.L. (2018). Combination Treatment with 5F9 and Azacitidine Enhances Phagocytic Elimination of Acute Myeloid Leukemia. Blood.

[26] Alfaro C., Teijeira A., Onate C., Perez G., Sanmamed M.F., Andueza M.P., Alignani D., Labiano S., Azpilikueta A., Rodriguez-Paulete A. (2016). Tumor-Produced Interleukin-8 Attracts Human Myeloid-Derived Suppressor Cells and Elicits Extrusion of Neutrophil Extracellular Traps (NETs). Clin. Cancer Res..

[27] Ott P.A., Hu Z., Keskin D.B., Shukla S.A., Sun J., Bozym D.J., Zhang W., Luoma A., Giobbie-Hurder A., Peter L. (2017). An immunogenic personal neoantigen vaccine for patients with melanoma. Nature.

[28] Sahin U., Derhovanessian E., Miller M., Kloke B.P., Simon P., Lower M., Bukur V., Tadmor A.D., Luxemburger U., Schrors B. (2017). Personalized RNA mutanome vaccines mobilize poly-specific therapeutic immunity against cancer. Nature.

